# Trisomy 18 and Trisomy 13: A Retrospective Cohort Study at a Tertiary Hospital

**DOI:** 10.3390/children13020271

**Published:** 2026-02-14

**Authors:** Nihan Uygur Külcü, Nurdan Erol, Sümeyra Oguz, Ayşenur Celayir, Güner Karatekin, Özge Yatır Alkan

**Affiliations:** 1 Department of Pediatrics, University of Health Sciences, Zeynep Kamil Maternity and Children’s Training and Research Hospital, 34668 Istanbul, Türkiye; 2 Department of Medical Genetics, University of Health Sciences, Zeynep Kamil Maternity and Children’s Training and Research Hospital, 34668 Istanbul, Türkiye; 3 Department of Pediatric Surgery, University of Health Sciences, Zeynep Kamil Maternity and Children’s Training and Research Hospital, 34668 Istanbul, Türkiye; 4 Department of Neonatology, University of Health Sciences, Zeynep Kamil Maternity and Children’s Training and Research Hospital, 34668 Istanbul, Türkiye

**Keywords:** Trisomy 18, Trisomy 13, Kaplan–Meier curve, survival analysis, neonatal mortality, congenital heart disease, nosocomial infection, intensive care unit

## Abstract

**Highlights:**

**What are the Main Findings?**

- Children with Trisomy 18 and Trisomy 13 exhibited very high mortality rates, particularly within the first six months of life, with severe cardiac anomalies and nosocomial infections being the leading causes of death.
- Survival analysis showed no statistically significant difference between Trisomy 18 and Trisomy 13, indicating that prognosis is influenced more by individual clinical factors than by trisomy type alone.
- A small but notable subgroup of patients survived for months to years, demonstrating that clinical trajectories are heterogeneous rather than uniformly fatal.

**What is the Implication of the Main Finding?**

- These findings challenge the traditional view that Trisomy 18 and Trisomy 13 are universally incompatible with life.
- Clinical management should move away from a uniform palliative approach toward individualized, multidisciplinary decision-making based on disease severity and comorbidity burden.
- Early infection prevention, careful cardiopulmonary management, and family-centered ethical counseling may improve survival and quality of care in selected patients.

**Abstract:**

Background: Trisomy 18 (T18; Edwards syndrome) and Trisomy 13 (T13; Patau syndrome) are rare autosomal aneuploidies characterized by severe congenital anomalies, high neonatal mortality, and complex clinical trajectories. Objective: This study aimed to describe the clinical features, management approaches, and outcomes of genetically confirmed patients aged 0–18 years diagnosed with T18 or T13 in a tertiary care center. Methods: This retrospective study reviewed hospital records of genetically confirmed T18 and T13 cases identified through ICD-10 codes (Q91–Q92) between January 2015 and December 2024. Patients aged 0–18 years at diagnosis were included. Demographic, clinical, and interventional data were collected from electronic medical records. Survival analyses were conducted using the Kaplan–Meier method, with comparisons assessed using the log-rank test. Results: Among 29 patients, 23 had T18 and 6 had T13. Cardiovascular involvement was the most frequent anomaly, and overall mortality was high despite intensive care. Median survival was 90 days for T18 and 120 days for T13, with more than 80% surviving the first month but showing a steep decline thereafter. Most deaths were attributed to cardiopulmonary complications or sepsis secondary to prolonged intensive care. Kaplan–Meier analysis revealed marked early mortality in both groups, with no significant survival difference (log-rank *p* ≈ 0.3). A small subset demonstrated longer-term survival with heterogeneous clinical courses. Conclusions: T18 and T13 are associated with high early mortality driven by complex congenital heart disease, respiratory instability, and infection-related complications. Although the overall prognosis remains poor, a minority of patients achieve extended survival, highlighting variable trajectories. Early multidisciplinary care, individualized decision-making, and strict infection prevention remain essential to optimize outcomes and support families.

## 1. Introduction

Trisomy 18 (T18–Edwards syndrome) and Trisomy 13 (T13–Patau syndrome) are the second and third most common autosomal trisomies after Trisomy 21 (T21–Down syndrome) [1]. Chromosomal abnormalities occur in approximately 1 in 140 to 1 in 153 live births [2,3]. Both conditions can be diagnosed prenatally or postnatally using karyotyping, fluorescence in situ hybridization (FISH), or chromosomal microarray analysis (CMA) [4]. According to postnatal karyotyping data from Türkiye, the prevalence of T18 is 12.6 per 10,000 live births and T13 is 4.9 per 10,000 live births—figures consistent with estimates based on clinical suspicion [5].

T13 and T18 are characterized by multiple congenital anomalies affecting the cardiovascular, central nervous, and gastrointestinal (GI) systems [6,7]. These anomalies contribute to high neonatal and infant mortality, while survivors frequently experience profound neurodevelopmental impairments, including global developmental delay, intellectual disability, and motor dysfunction [8,9]. For clinicians and families, understanding the expected clinical trajectory is essential when making decisions about resuscitation, surgical interventions, and the level of intensive care support and hospital readmissions in milder cases discharged home [10,11].

Recent reports indicate that aggressive or individualized management may extend survival in selected cases, challenging the historical perception of these syndromes as uniformly “incompatible with life” [12,13]. Additionally, early integration of pediatric palliative care and family-centered planning has facilitated more ethically grounded and personalized care pathways [14].

Despite these advances, survival patterns remain highly variable, and contemporary real-world data—particularly from diverse healthcare systems—are limited. Therefore, the aim of this study was to characterize the clinical features, management strategies, and survival outcomes of children with T13 and T18 treated at a tertiary-level specialty hospital over the past decade.

## 2. Materials and Methods

### 2.1. Study Setting and Clinical Procedures

This study was conducted at a tertiary-level specialty hospital, providing services in Obstetrics and Gynecology, Perinatology, and Pediatrics. The hospital includes a Genetic Diagnosis and Screening Center, a Level IV Neonatal Intensive Care Unit (NICU), a Pediatric Surgery Department, a Pediatric Surgical Intensive Care Unit (SICU) for neonates requiring surgical intervention, and a Pediatric Intensive Care Unit (PICU) for children beyond the neonatal period. Pediatric and neonatal patients requiring cardiovascular surgery were referred to a nearby specialized Cardiovascular Surgery Center, and post-discharge care was provided through general and subspecialty pediatric outpatient clinics.

For pregnancies with a prenatal diagnosis of Trisomy 13 or Trisomy 18, termination of pregnancy is offered as a standard option. If the family consents, the procedure is performed in accordance with institutional and national regulations. When termination is declined, expectant mothers are referred to a tertiary-level center with neonatal and pediatric surgical services to ensure a multidisciplinary approach to perinatal management and delivery.

### 2.2. Study Design and Patient Selection

This retrospective study analyzed International Statistical Classification of Diseases and Related Health Problems, 10th Revision (ICD-10) codes—specifically ICD-Q91 (T18) and ICD-Q92 (T13), including their sub-codes—from the hospital database for patients aged 0–18 years at the time of diagnosis, covering the period from January 2015 to December 2024. A total of 43 cases were initially identified. Of these, 23 patients with T18 and 6 with T13 formed the final study cohort. Fourteen cases were excluded: six had normal karyotypes despite clinical suspicion, three had other syndromic or chromosomal abnormalities, three represented abortion materials diagnosed with T13/T18, and two had incomplete records. Only genetically confirmed cases with complete datasets were included to minimize selection and information bias. The study was approved by the institutional Research Ethics Committee (Approval number: 19.03.2025/43).

### 2.3. Data Collection and Variables

A standardized data extraction protocol was applied, and information was obtained from electronic medical records, karyotype results, and genetic testing reports. The following parameters were collected: sex, survival status (alive or deceased), maternal age, gestational age (weeks), maternal comorbidities, type of delivery, birth weight, intrauterine growth retardation/small for gestational age (IUGR/SGA), age at admission, admission and hospitalization departments (outpatient clinics, NICU, PICU, pediatric surgery), age at death, age at last follow-up for survivors, and hospital stay duration. Demographic data, systemic examination findings, and major interventions were also reviewed.

### 2.4. Statistical Analysis

Statistical analyses were performed using IBM SPSS Statistics version 22.0 (IBM Corp., Armonk, NY, USA). Continuous variables were expressed as mean ± standard deviation (SD) and median (minimum–maximum), whereas categorical variables were presented as counts (n) and percentages (%). Survival outcomes were analyzed using the Kaplan–Meier method, and survival distributions between the T18 and T13 groups were compared using the log-rank test.

ICU length of stay was analyzed on an admission-based level because several patients had multiple ICU admissions during their clinical course. Each ICU admission was counted as a separate event. Due to the limited sample size, no multivariate analyses or regression modeling were conducted, and confidence intervals were not estimated. A *p*-value < 0.05 was considered statistically significant.

## 3. Results

A total of 29 patients were identified, including 23 with T18 and 6 with T13. Antenatal diagnosis was established in six cases (four T18 and two T13), while the remaining patients were diagnosed postnatally through karyotype or CMA. One T13 patient had mosaicism and was excluded from the primary survival analysis due to a mild phenotype. Table 1 summarizes the baseline demographic and perinatal characteristics of all patients.

As shown in Table 2, both Trisomy 18 and Trisomy 13 cases required high rates of intensive neonatal care, with substantial differences observed in survival duration and ICU stay.

**Figure 1:**
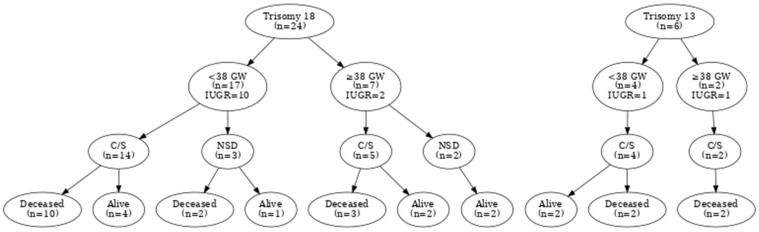
Flowcharts of patients with Trisomy 18 and Trisomy 13 cases showing gestational age, intrauterine growth restriction (IUGR), type of delivery, and current status (alive/deceased).

Among all cases, twenty infants were inborn and admitted to the NICU immediately after birth. Two outborn and eight inborn patients required admission to the SICU, while four outborn and four inborn patients who had surpassed the neonatal period were transferred to the PICU for continued intensive care support. The overall cesarean section rate was 82.8%, with rates of 78.2% in T18 and 100% in T13. Among T18 infants delivered before 38 gestational weeks, 72.2% were born via cesarean section. Figure 1 illustrates the distribution of delivery mode, gestational age, IUGR, and survival status for both trisomy groups.

Cardiovascular findings were absent in two T13 patients with normal echocardiography; echocardiographic documentation was incomplete in two deceased cases. Table 3 provides a detailed overview of cardiovascular anomalies. Five patients required cardiac intervention at a specialized pediatric cardiovascular center: two underwent pulmonary artery banding with Patent Ductus Arteriosus (PDA) ligation, one had complete tetralogy of Fallot repair, one received a ductal stent, and one had a pacemaker implanted. Only one patient who underwent pulmonary banding and PDA ligation survived and remained hospitalized in the ICU at last follow-up.

GI anomalies were identified in ten patients, all of whom were referred to pediatric surgery shortly after birth. Additional systemic anomalies and organ involvement are listed in Table 4. Four infants underwent gastrostomy tube placement, of whom two survived. Three patients with cleft palate received orogastric feeding with high-calorie formulas, and two patients with intestinal atresia underwent colostomy. A total of five patients required tracheostomy; two survived to discharge, although both remained oxygen-dependent.

Survival outcomes are summarized in Table 1. The median survival time was 90 days for T18 and 120 days for T13. One-month survival exceeded 80% in both groups; by six months, survival declined to 34.8% for T18 and 60% for T13, and by one year to 26.1% and 40%, respectively.

In T18, survival ranged from 1 to 4190 days, with an overall mortality rate of 69.6%, 87.5% of which occurred within the first six months of life. In T13 cases, survival ranged from 13 to 1380 days, with a mortality rate of 50%.

Hospitalization burden varied across survival intervals. When stratified by survival at 1 month, 6 months, and 1-year, cumulative hospital stay differed between subgroups, with the highest inpatient burden among infants surviving beyond the neonatal period. Hospitalization duration is presented separately for each interval, providing a more granular view of care intensity over time (Table 5).

The characteristics of surviving patients with T18 and T13 are presented in Table 5.

**Figure 2:**
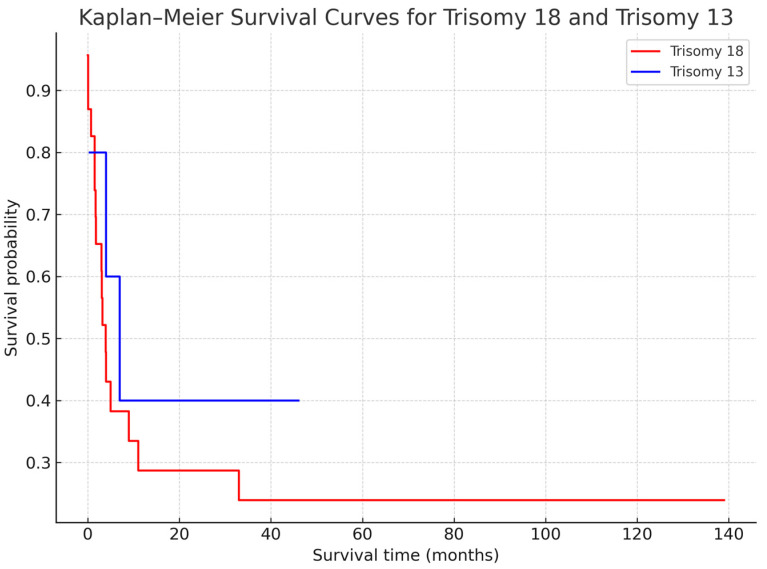
Kaplan–Meier survival curves for patients with Trisomy 18 (red) and Trisomy 13 (blue). Both syndromes showed a high mortality rate during the early postnatal period, with survival probabilities stabilizing among a small subset of long-term survivors. Numbers at risk for each time interval are displayed below the graph. No statistically significant survival difference was detected between the two groups (log-rank *p* ≈ 0.3).

Kaplan–Meier analysis demonstrated steep early postnatal mortality followed by a plateau among long-term survivors for both groups. No statistically significant difference was detected between the T18 and T13 survival curves (log-rank *p* ≈ 0.3). Kaplan–Meier survival curves for both trisomy groups are shown in Figure 2.

Overall, nine deaths were attributed to sepsis secondary to nosocomial infections (one due to candidal septicemia and eight due to Gram-negative bacteremia). The remaining deaths were primarily caused by cardiopulmonary complications, including progressive heart failure and respiratory insufficiency. Four patients underwent cardiac surgery; only one survived but remained ICU-dependent at last evaluation. Mortality overall was predominantly associated with severe congenital heart disease (CHD), secondary pulmonary hypertension, and infection-related complications.

Because some patients required repeated ICU admissions, ICU stay was evaluated on an admission-based rather than patient-based level. This approach better reflects the intensive care burden across the cohort. A total of 39 ICU admissions were recorded among patients with T18 and T13. Admission-based mean ICU stay was 52.3 (2–210) days. Among ICU admissions of deceased patients (n = 24), the mean length of stay was 57.4 (6–180) days. Among ICU admissions of surviving patients (n = 15), the mean ICU stay was 44.1 (2–210) days.

The cohort initially comprised 23 children with trisomy. Hospitalization burden was analyzed in consecutive age intervals, including only children who were alive at the beginning of each interval (Table 6). During the first 30 days of life, 3 children died at 2, 10, and 22 days of age. These three children accounted for a total of 34 inpatient days. Four children had no hospital admissions during this period. The remaining 16 children were hospitalized in the neonatal intensive care unit and accumulated 480 inpatient days. Overall, the total hospitalization burden during the first 30 days was 514 inpatient days.

At 3 months of age, 13 children remained alive; 10 had died during the first 3 months. Among the 23 children initially included, 3 had no hospital admissions during this period, while the remaining 20 children accumulated a total of 1247 inpatient days. Thirteen children survived beyond 3 months and entered the 3–6-month interval. During this period, 5 children died. Of these 13 children, 6 had no hospitalizations, whereas 7 children accounted for a total of 316 inpatient days; two of them required prolonged hospitalization (90 days each). Eight children survived to 6 months of age. Between 6 months and 1 year, 2 children died, and 4 children accumulated a total of 269 inpatient days. Six children survived beyond 1 year, at which point follow-up for the present analysis ended.

## 4. Discussion

T18 and T13 are severe chromosomal anomalies associated with significant perinatal and neonatal morbidity and mortality [6,12]. Because of their clinical and epidemiological impact, both conditions are actively monitored by the European Concerted Action on Congenital Anomalies and Twins (EUROCAT) [15]. Infants with these diagnoses frequently require prolonged neonatal intensive care and multiple surgical interventions, reflecting their complex clinical course.

Only six of the 29 patients (20.7%) in our series had an antenatal diagnosis, a proportion that can be explained by several contextual factors. The study included only live-born or postnatally confirmed cases from a tertiary center, where most diagnoses were made after delivery. Moreover, during the earlier study years, the availability and use of non-invasive prenatal testing (NIPT) and CMA were limited in our region. Finally, some families declined invasive prenatal testing despite suggestive ultrasonographic findings, largely due to cultural or ethical considerations.

Advanced maternal age (>35 years), a well-established risk factor for T18 and T13 [16], was observed in 50% of T18 and 80% of T13 pregnancies in our cohort. Reported cesarean section (C/S) rates in the literature range from 48% to 90%, primarily due to fetal or maternal indications and high rates of preterm delivery [6,17]. The C/S rate in our cohort (78% for T18 and 100% for T13) was comparable to these reports. IUGR was more frequent in our population than previously reported, likely reflecting rigorous fetal surveillance and improved detection accuracy. These obstetric patterns reinforce the severity of fetal anomalies and institutional delivery practices.

Moreover, early preterm (<32 weeks) and small-for-gestational-age (SGA) infants with congenital anomalies have a higher risk of chromosomal trisomies (10.8% vs. 2.3%) [18]. In our cohort, neonatal mortality reached 66.7% in early preterm IUGR infants and 100% in term IUGR infants, underscoring the prognostic significance of fetal growth restriction in this population. These findings highlight the importance of early genetic screening in SGA infants to enhance diagnostic precision and guide perinatal management strategies.

Recent multicenter studies have shown that T18 and T13 can no longer be described as “incompatible with life”. Although early postnatal mortality remains high, a small but meaningful subset of patients survive months to years, with reported survival extending into late childhood in selected cases [19,20]. This paradigm shift emphasizes that prognosis should be presented on a spectrum rather than as a uniform fatal condition. In our series, survival durations among the six surviving T18 patients ranged from 4 to 11.5 months, aligning with this heterogeneous survival pattern.

GI anomalies are hallmark features of T18, with reported rates of esophageal atresia ranging from 16% to 18% and omphalocele or diaphragmatic hernia occurring in fewer than 10% of cases [6]. Nearly all T18 infants admitted to the pediatric surgery intensive care unit in our series had GI involvement, emphasizing the importance of genetic evaluation in dysmorphic neonates presenting with major GI malformations. Prematurity, low birth weight, and prolonged ICU stays further increased susceptibility to infections [6]; none of nine infants in our cohort with Gram-negative or candidal septicemia survived.

Cardiac anomalies were universal among T18 patients in our cohort, consistent with the literature documenting near-universal CHD in this population [21]. Cardiac involvement was generally less severe in survivors than in deceased individuals. Most surgical interventions were deferred because of fragile clinical status and multiple coexisting anomalies. Septicemia-related complications resulted in death in all four patients referred for cardiac surgery. Among T13 patients, structural CHD was confirmed in 2 of 6 cases (33.3%); echocardiographic data were unavailable for two deceased patients, so the true prevalence may be higher.

The marked variability observed in our cohort reflects the heterogeneous nature of T13 and T18, where prognosis is strongly influenced by gestational age, birth weight, comorbidity burden, infection risk, and access to surgical or intensive care interventions. Current literature recommends individualized rather than protocol-driven decision-making for infants with these conditions, recognizing that management must be tailored to each infant’s clinical condition and overall malformation load [20,22].

The impact of surgical intervention on survival remains controversial [10,21,23]. Some studies suggest improved outcomes among selected patients undergoing cardiac or gastrointestinal surgery in the neonatal period [10,23]. Greene reported significantly higher one-year survival rates among infants who underwent cardiac repair compared with those who did not [24]. Similarly, Ma et al. showed that nearly 90% of T18 patients survived to discharge after cardiac surgery [25]. However, other authors argue that aggressive surgical intervention may prolong survival without achieving meaningful long-term benefit, particularly when such procedures delay palliative decision-making [21].

In contrast to published reports, all patients referred for cardiac surgery in our cohort died. This likely reflects the predominance of palliative—not corrective—procedures, advanced disease severity, and high comorbidity burden. Predictive models have indicated that cardiac repair, gastrostomy placement, and prolonged ventilatory support are among the strongest contributors to short-term survival [26]. Nonetheless, standardized criteria for surgical candidacy remain lacking, and clinical decisions must rely on individualized assessments.

Although cardiac surgery was performed in a small subset of our cohort, postoperative survival was ultimately limited by non-cardiac causes, reflecting the dominant impact of multisystem disease in trisomy 13 and 18. The present study was not designed to determine which specific cardiac defects derive the greatest benefit from surgical versus conservative management. Defect-specific recommendations require larger, preferably multicenter cohorts with detailed perioperative and long-term follow-up. Future studies integrating cardiac anatomy, extracardiac disease burden, and postoperative trajectories are needed to define which lesions may be amenable to intervention in a way that meaningfully alters both survival and care burden.

Cortezzo et al. reported that T13 diagnosis, very low birth weight (<1000 g), and adoption of comfort care were associated with reduced survival [27]. Conversely, another study found no significant impact of family decisions on outcomes [28], highlighting variability in institutional policies across countries.

In our hospital, families of infants diagnosed with T13 or T18 are informed immediately after birth about the newborn’s condition. Under current national regulations, families do not have legal authority to request withholding or withdrawal of life-sustaining treatments. All medically indicated interventions are performed, and families generally consent to necessary procedures. When surgical procedures involving cardiovascular or other organ systems are needed, patients are managed at our pediatric surgery unit or a nearby cardiovascular surgery center and subsequently monitored in the NICU and PICU. Children with milder clinical manifestations may be considered for home-based care with gastrostomy, tracheostomy, or home mechanical ventilation. Families unable to provide such care are referred to social services. All families receive genetic counseling regarding recurrence risks and future pregnancy planning.

In a large, population-based, multi-registry analysis by Goel et al. including 16 registries with complete 1-year follow-up, first-year mortality reached 87% for T13 and 88% for T18. Both prevalence and survival outcomes demonstrated substantial variation across countries, underscoring the influence of regional practices, healthcare systems, and cultural approaches to care on postnatal management and prognosis [29]. In our study, the median survival time for T18 exceeded the classical averages reported in the literature (7–14 days) [30]. This extended survival likely reflects the active supportive management routinely practiced in our institution. The presence of infants who survived several months reinforces the notion that T18 exhibits broad variability in clinical trajectory. Similar findings in the recent literature support the recognition of long-term survivors as part of the clinical spectrum [30].

Gastrointestinal surgical interventions, postoperative feeding difficulties, growth failure, and infection-related complications represent major determinants of outcome in trisomy 13 and 18. Although these factors were present in our cohort, they could not be analyzed in sufficient detail within the scope of this file-based study. A comprehensive evaluation of gastrointestinal surgery, nutritional trajectories, postoperative challenges, and their impact on survival and quality of life is currently being conducted by the pediatric surgery team and will be reported separately.

In the absence of standardized quality-of-life instruments, clinical indicators such as hospitalization burden, ICU exposure, and dependence on respiratory or nutritional support serve as pragmatic proxies for daily life impact in this population. In our cohort, long-term survivors often required prolonged inpatient care and ongoing medical support after discharge, highlighting the substantial caregiving burden on families.

These findings underscore the need for individualized, multidisciplinary case management involving neonatology, genetics, cardiology, pediatric surgery, nutrition, and palliative care teams, with early goal-setting, realistic prognostic counseling, and structured discharge planning.

We acknowledge the limitations of our prenatal data and emphasize that future prospective, multicenter studies with standardized imaging parameters are needed to clarify how specific ultrasound findings relate to postnatal survival and long-term care trajectories, enabling more precise prenatal counseling.

This study has limitations, including its retrospective design, single-center setting, and small sample size, which may reduce generalizability. Potential information bias may have arisen due to varying follow-up durations and reliance on medical records. Institutional practices and sociocultural factors may also influence outcomes and limit broader applicability.

Given the variability in disease progression and improvements in neonatal and surgical care, further multicenter studies and standardized clinical guidelines are needed. Priorities for future research include developing national registries, refining criteria for surgical candidacy, and implementing early genetic screening strategies for high-risk infants such as those with SGA. Ultimately, improved outcomes for infants with T18 and T13 will rely on ethically grounded, multidisciplinary, and individualized clinical care.

## 5. Conclusions

Although overall survival for infants with Trisomy 18 and Trisomy 13 remains poor, our findings indicate that survival can be prolonged in selected cases receiving active, multidisciplinary care. Infection control and early management of cardiopulmonary complications appear to play decisive roles in extending life beyond the neonatal period. These observations emphasize the importance of individualized clinical decisions rather than a uniform palliative approach for all patients.

Despite high neonatal mortality and complex medical requirements, some infants may benefit from tailored interventions, intensive monitoring, and family-centered care. Early genetic diagnosis, multidisciplinary collaboration, and compassionate, ethically guided decision-making remain essential to improving outcomes. Establishing national registries, developing standardized treatment guidelines, and expanding access to prenatal and neonatal screening programs represent key future priorities for optimizing care in this challenging population.

Prolonged hospitalization and medical uncertainty place a substantial psychological burden on families, often resulting in sustained anxiety and emotional exhaustion. Integrating structured psychosocial support, parental counseling, and palliative care expertise into routine management should therefore be regarded as a core component of care and a public health priority.

## Figures and Tables

**Table 1:** The demographic and perinatal characteristics of Trisomy 18 and Trisomy 13 patients.

	Trisomy 18	Trisomy 13	Total
	n = 23, n (%)	n = 6, n (%)	n = 29, n (%)
**Sex**			
*Female*	18 (78.3)	2 (33.3)	20 (69.0)
*Male*	5 (21.7)	4 (66.7)	9 (31.0)
**Birthweight (g)**	1790	2170	1810
	(1110–2400)	(1500–2770)	(1110–2770)
**Birthweight Classification**			
*1000–1499*	3 (13.0)	0 (0.0)	3 (10.4)
*1500–2500*	20 (87.0)	4 (66.7)	24 (82.8)
*>2500*	0 (0.0)	2 (33.3)	2 (6.8)
**Gestational age**			
*<32 weeks*	11 (47.9)	2 (33.3)	13 (44.8)
*33–37 weeks*	5 (21.7)	3 (50.0)	8 (27.6)
*>38 weeks*	7 (30.4)	1 (16.7)	8 (27.6)
**Mother’s age (years)**	35 (20–42)	35 (24–38)	35 (20–42)
**Mother’s age classification**			
*18–34 years*	11 (47.9)	2 (33.3)	13 (44.8)
*≥35 years*	12 (52.1)	4 (66.7)	16 (55.2)
**Presence of maternal comorbidity**			
Polyhydramnios	3 (13.0)	1 (16.7)	4 (13.7)
Preeclampsia	2 (8.7)	0 (0.0)	2 (6.8)
GDM	2 (8.7)	0 (0.0)	2 (6.8)
Hypothyroidism	1 (4.3)	0 (0.0)	1 (3.4)
Thalassemia	1 (4.3)	0 (0.0)	1 (3.4)
STD	1 (4.3)	0 (0.0)	1 (3.4)
Single umbilical artery	0 (0.0)	1 (16.7)	1 (3.4)
Meconium-stained amniotic fluid	0 (0.0)	1 (16.7)	1 (3.4)
**Type of delivery**			
*C/S*	18 (78.2)	6 (100)	24 (82.8)
*NSD*	5 (21.8)	0	5 (11.2)
**IUGR-SGA**	11 (47.9)	3 (50.0)	14 (48.3)

**Abbreviations:** C/S: Cesarean Section, GDM: gestational diabetes mellitus; IUGR: intrauterine growth retardation; SGA: small for gestational age; STD: Sexually transmitted disease.

**Table 2:** Survival outcomes and postnatal characteristics of infants with trisomy 13 and trisomy 18.

	Trisomy 18	Trisomy 13	Total
	n = 23, n (%)	n = 6, n (%)	n = 29, n (%)
**Age on admission**			
*At birth*	15 (65.2)	4 (66.7)	19 (65.6)
*0–28 days*	2 (8.7)	0 (0.0)	2 (6.8)
*>28 days*	6 (26.1)	2 (33.3)	8 (27.6)
**Admission and hospitalization place**			
*NICU*	18 (78.3)	2 (33.3)	20 (68.9)
*Pediatric surgery*	8 (34.8)	2 (33.3)	10 (34.5)
*PICU*	6 (26.1)	2 (33.3)	8 (27.6)
*Outpatient pediatric clinics*	4 (17.4)	3 (50.0)	7 (24.1)
**Status**			
*Deceased*	17 (73.9)	3 (50)	20 (68.9)
*Alive*	6 (26.1)	3 (50)	9 (31.1)
**Median survival time (days)**	90 (1–4190)	120 (13–1380)	(1–4190)
**1st month survival**	19 (82.6)	4 (80)	23 (79.3)
**6th month survival**	8 (34.8)	3 (60)	11 (37.9)
**One year survival**	6 (26.1)	2 (40)	8 (27.6)
**Mean ICU stay (days)**	82 (8–210)	16.9 (2–48)	52.3 (2–210)

**Abbreviations:** NICU: neonatal intensive care unit; PICU: pediatric intensive care unit.

**Table 3:** Characteristics of Trisomy 18 and Trisomy 13 patients with cardiovascular involvement.

	Pathology	Type	n (%)
Trisomy 18 (n = 23)			
**Deceased (n = 16)**	VSD ± BAV ± ASD ± PDA	Inlet (large)	**6 (37.5)**
		Inlet-outlet (large)	**2 (12.5)**
		Pm,pm-outlet (large)	**3 (18.8)**
		Muscular (large)	**3 (18.8)**
		Pm (moderate) + MS	**1 (6.3)**
	Unbalanced AVSD + PS+ Pulmonary hypoplasia		**1 (6.3)**
	DORV (Fallot type)		**1 (6.3)**
**Alive (n = 7)**	VSD ± BAV ± ASD ± PDA	Muscular (small)	**1 (14.3)**
		Pm (intermediate) + AS + PS	**1 (14.3)**
		Pm,Outlet (large)	**3 (42.9)**
		Inlet (large) + arcus hypoplasia	**1 (14.3)**
	BAV + Arcus anomaly		**1 (14.3)**
**Trisomy 13 (n = 6)**			
**Alive (n = 3)**	Normal ECHO		**2 (66.7)**
	PDA +ASD (small)		**1 (33.3)**
**Deceased (n = 3)**	Fallot + severe PS		**1 (33.3)**
	**Unknown**		**2 (66.7)**

**Abbreviations:** AS: Aortic stenosis, ASD: Atrial septal defect, AVSD: Atrioventricular septal defect, BAV: Bicuspid aortic valve, DORV: Double outlet right ventricle, ECHO: Echocardiography, MS: Mitral stenosis, PDA: Patent ductus arteriosus, Pm: perimembranous, PS: pulmonary stenosis, VSD: Ventricular septal defect.

**Table 4:** Clinical features of Trisomy 18 patients (n = 23).

Involvement		n	%
**Abdominal and gastrointestinal system**	Omphalocele	**3**	**13.0**
	Esophageal atresia (EA) (n = 2 with TOF)	**3**	**13.0**
	Intestinal atresia	**2**	**3.1**
	Meckel diverticulitis	**1**	**4.3**
	Diaphragmatic hernia	**2**	**3.1**
	Umbilical hernia	**1**	**4.3**
	Inguinal hernia	**4**	**17.4**
**Neurologic**	Epilepsy	**4**	**17.4**
	Structural CNS anomalies: Holoprosencephaly, corpus callosum agenesis, mega cisterna magna, vermis hypoplasia	**12**	**52.2**
**Craniofacial region**	Cleft palates and lips	**3**	**13.0**
**Musculoskeletal**	**Hand & feet** anomalies	**9**	**39.1**
	Syndactyly-polydactyly	**9**	**39.1**
**Ocular**	Anophthalmia, Retinal detachment, Bilateral fibrotic bands, Loss of vision	**3**	**13.0**
**Urinary system**	Nephrolithiasis:1 Hydro-ureteronephrosis:2	**3**	**13.0**
**Endocrinologic**	Undescended testes:3 Hypothyroidism:2 Adrenal insufficiency:1	**4**	**17.4**

**Abbreviations:** CNS: central nervous system, EA: esophageal atresia, TOF: tracheoesophageal atresia.

**Table 5:** Characteristics of surviving patients with Trisomy 18 and Trisomy 13.

Case	Trisomy Type	Sex	Birth Date	Postnatal Age (Months)	Type of Delivery	Gestational Age (GW)	Birth Weight (g)	Involvement Affecting Life Quality	ECHO	ICU Stay (Days)	Medical Interventions	Epilepsy	CVS Operation
**1**	T18	F	2015	139	C/S	37	1400	Neurologic, Renal	VSD PDA	128		+	
**2**	T18	F	2014	128	C/S	38	1810	CVS, Neurologic	VSD PAH	57		+	
**3**	T18	M	2018	73	C/S	35	1560	CVS, Neurologic, Endocrinologic, Respiratory	ASD VSD PAH	350	Tracheostomy PEG	+	
**4**	T18	F	2019	68	NSD	38	2400	Neurologic	ASD, Bicuspid Ao, Atrial Septal Aneurysm	7		Mental retardation	
**5**	T18	F	2024	4	C/S	32	1910	CVS, Neurologic Respiratory (* still hospitalized in PICU)	ASD VSD Aortic Hypoplasia, Bicuspid Aortic Valve, Small Ductus Arteriosus	125	Tracheostomy PEG	+	Pulmonary artery banding, PDA ligation
**6**	T18	F	2021	38	C/S	38	1880	CVS, Neurologic	VSD, PAH		OGT feeding	+	
**7**	T13	F	2022	29	C/S	36	2000	Neurologic	ASD, PAH, peripheral pulmonary stenosis	41	PEG	+	
**8**	T13	M	2021	46	C/S	37	2680	Respiratory Renal, Neurologic, Endocrine	ASD	28	PEG, tracheostomy (home vent)	+	
**9**	T13, mosaicism	M	2006	216	C/S	38	2600	Renal Endocrine Orthopedic	N	Unknown			

* Patient initials have been removed to protect confidentiality. +: Indicates presence of the relevant condition or intervention. **Abbreviations:** ASD: Atrial Septal Defect, Ao: Aorta, C/S: Cesarean Section, CVS: Cardiovascular System, ECHO: Echocardiography, F: Female, GW: Gestational Week, ICU: Intensive Care Unit (neonatal/pediatric/pediatric surgery), M: Male, NSD: Normal Spontaneous Delivery, OGT: Orogastric Tube, PAH: Pulmonary Arterial Hypertension, PDA: Patent Ductus Arteriosus, PEG: Percutaneous Endoscopic Gastrostomy, PICU: Pediatric Intensive Care Unit, T13: Trisomy 13, T18: Trisomy 18, VSD: Ventricular Septal Defect.

**Table 6:** Age-stratified hospitalization burden among children alive at the beginning of each interval.

Age Interval	Children at Risk (n)	Deaths (n)	Never Hospitalized (n)	Hospitalized (n)	Total Inpatient Days	Notes
0–30 days	23	3	4	19	514	3 deaths at days 2, 10, and 22; deceased children contributed 34 inpatient days
0–3 months	23	10	3	20	1247	Cumulative burden during first 3 months
3–6 months	13	5	6	7	316	Two children hospitalized for 90 days each
6–12 months	8	2	–	4	269	–
≥12 months	6	–	–	–	–	

–: Indicates no hospitalization. *Only children alive at the beginning of each age interval were included in that interval’s analysis. Inpatient days include all hospital stays occurring within the specified age period.*

## Data Availability

The data supporting the findings are available from the corresponding author upon reasonable request.

## Abbreviations

The following abbreviations are used in this manuscript: AS, Aortic Stenosis; ASD, Atrial Septal Defect; AVSD, Atrioventricular Septal Defect; BAV, Bicuspid Aortic Valve; C/S, Cesarean Section; CHD, Congenital Heart Disease; CMA, Chromosomal Microarray Analysis; CNS, Central Nervous System; CVS, Cardiovascular System; DORV, Double Outlet Right Ventricle; EA, Esophageal Atresia; ECHO, Echocardiography; EUROCAT, European Concerted Action on Congenital Anomalies and Twins; FISH, Fluorescence In Situ Hybridization; GDM, Gestational Diabetes Mellitus; GI, Gastrointestinal; GW, Gestational Week; ICU, Intensive Care Unit; IUGR, Intrauterine Growth Retardation; NICU, Neonatal Intensive Care Unit; NIPT, Non-Invasive Prenatal Testing; NSD, Normal Spontaneous Delivery; OGT, Orogastric Tube; PAH, Pulmonary Arterial Hypertension; PDA, Patent Ductus Arteriosus; PEG, Percutaneous Endoscopic Gastrostomy; PICU, Pediatric Intensive Care Unit; PS, Pulmonary Stenosis; SGA, Small for Gestational Age; SICU, Surgical Intensive Care Unit; T13, Trisomy 13; T18, Trisomy 18; T21, Trisomy 21; VSD, Ventricular Septal Defect.
